# Influence of freeze-thaw events on carbon dioxide emission from soils at different moisture and land use

**DOI:** 10.1186/1750-0680-2-2

**Published:** 2007-02-19

**Authors:** Irina Kurganova, Robert Teepe, Norman Loftfield

**Affiliations:** 1 Institute of Physicochemical and Biological Problems in Soil Science, Russian Academy of Sciences, Pushchino, Moscow region, 142290, Russia; 2 Institute of Soil Science and Forest Nutrition, Georg-August University Göttingen; Büsgenweg 2, 37077, Göttingen, Germany

## Abstract

**Background:**

The repeated freeze-thaw events during cold season, freezing of soils in autumn and thawing in spring are typical for the tundra, boreal, and temperate soils. The thawing of soils during winter-summer transitions induces the release of decomposable organic carbon and acceleration of soil respiration. The winter-spring fluxes of CO_2 _from permanently and seasonally frozen soils are essential part of annual carbon budget varying from 5 to 50%. The mechanisms of the freeze-thaw activation are not absolutely clear and need clarifying. We investigated the effect of repeated freezing-thawing events on CO_2 _emission from intact arable and forest soils (Luvisols, loamy silt; Central Germany) at different moisture (65% and 100% of WHC).

**Results:**

Due to the measurement of the CO_2 _flux in two hours intervals, the dynamics of CO_2 _emission during freezing-thawing events was described in a detailed way. At +10°C (initial level) in soils investigated, carbon dioxide emission varied between 7.4 to 43.8 mg C m^-2^h^-1 ^depending on land use and moisture. CO_2 _flux from the totally frozen soil never reached zero and amounted to 5 to 20% of the initial level, indicating that microbial community was still active at -5°C. Significant burst of CO_2 _emission (1.2–1.7-fold increase depending on moisture and land use) was observed during thawing. There was close linear correlation between CO_2 _emission and soil temperature (R^2 ^= 0.86–0.97, P < 0.001).

**Conclusion:**

Our investigations showed that soil moisture and land use governed the initial rate of soil respiration, duration of freezing and thawing of soil, pattern of CO_2 _dynamics and extra CO_2 _fluxes. As a rule, the emissions of CO_2 _induced by freezing-thawing were more significant in dry soils and during the first freezing-thawing cycle (FTC). The acceleration of CO_2 _emission was caused by different processes: the liberation of nutrients upon the soil freezing, biological activity occurring in unfrozen water films, and respiration of cold-adapted microflora.

## Background

The repeated freeze-thaw events during cold season, freezing of soils in autumn and thawing in spring are typical for the tundra, boreal, and temperate soils [1-5]. The winter-spring fluxes of CO_2 _from permanently and seasonally frozen soils are essential part of annual carbon budget varying from 5 to 50% and they should be not ignored [6-16].

Global warming scenarios predict a milder winter in high and middle latitude regions [17]. Changes in the over-winter temperature regime, including frequency of freezing-thawing and snowmelt processes will be more pronounced in open cropland in comparison with forest or grassland soils [18-20]. The freezing-thawing of soils induces the release of decomposable organic carbon, affects the composition and function of microbial communities, and thus has a profound influence on the overall functioning of ecosystems [21,22]. It was found that the decomposition of organic matter increased 10 times upon thawing in the forest soils of North America, which remained frozen most of the year [23]. In arctic soils, the carbon losses caused by repeated FTC amounted to 20–30 g C m^-2 ^during non-growing season [22].

The significant acceleration of soil respiration during thawing is well known [16,23-30]. The most common explanation for the increasing of respiratory activity upon freeze-thaw events is that the soil microbes are killed, releasing nutrient into the soil [31-33]. It was shown that a single FTC may kill up to 50% of the microbial biomass [16]. Later Herrmann and Witter [33] reported that only 5% of microbial biomass is destroyed during freezing-thawing, but this contributes 65% to the total C-flush. Using the ^14^C labelling plant residues and glucose, Kuzyakov and Sapronov [34] showed that the significant part of CO_2 _flush after thawing originated from root respiration (80% and 46% of total CO_2 _efflux from arable and forest soil, respectively), and the contribution of C from microbial biomass killed by freezing was the second important source of C-flush. Substantial over-winter losses of aboveground materials from various catch and cover crops have also been found [30,35,36]. The freeze-thaw-released organic C from microbes and plants are readily available for living microorganisms and may play the significant role in freeze-thaw-induced N_2_O emission [30,37-39]. Some mathematical models are developed to simulate the observed activation dynamics of gas emission, which account the burst of microbial growth on nutrients released into soil from frost destroyed cells [11,40]. Moreover, in recent study of Koponen *et al. *[3] it was shown that freezing and thawing of boreal soils does not have a strong effect on microbial biomass and structure of microbial community. Thus, the mechanisms of freeze-thaw activation are not absolutely clear and need future clarification.

The soil moisture is a key factor regulating the respiratory activity of soil [41]. Land use of soil has a profound effect on CO_2 _emission rate as well, governing the main soil properties (C and N content, pH, microbial activity, structure *etc*). However, the influence of soil moisture and land use on the freezing-thawing-induced CO_2 _emission is still poorly studied. For instance, Koponen and Martikainen [2] did not find the significant difference in freeze-thaw-released CO_2 _from soils at moisture corresponding to 56% and 85% of water filled pore space (WFPS). The influence of site location and land use were investigated by Prieme and Christensen [27] and Dorsch *et al. *[20]. They showed that total surplus of CO_2 _emission from grassland and fallow sites following thawing was generally higher than from arable and forest sites probably due to decomposition of carbon sources liberated from stressed grass roots [27] and residual plant activity at low temperature [20].

In this article we try to display how the soil moisture and land use affect CO_2 _production during repeated FTC. The present study was aimed to investigate the dynamics of CO_2 _emission from intact arable and forest soils (Luvisols, loamy silt; Low Saxony, Germany) at different water content during two FTC. The "dry" soil corresponds to 65% of WHC, and "wet" soil corresponds to 100% of WHC. We have chosen these contrast levels of soil moisture to simulate the natural soil conditions during cold season after the dry and wet autumn.

## Results

### Duration of soil freezing and thawing

Our investigation showed that the pattern of soil freezing and thawing depended on soil moisture, land use, and temperature during the freezing period (deep of frost). Thus, the duration of soil freezing varied from 1.3 to 2.7 days and the order of precedence was: ADS < FDS < AWS < FWS. We also found that the time to thaw the soils was shorter than to freeze and changed between 0.9–1.6 days. During the second cycle, the duration of freezing and thawing was shorter due to more mild frost (-3°C) compared to the first one (-5°C).

### CO_2 _dynamics during freezing-thawing cycles

**Figure 1:**
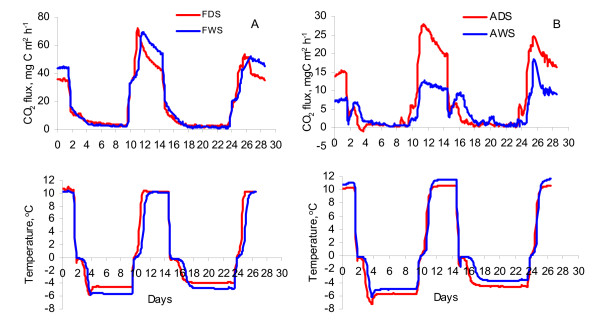
Dynamics of CO_2_ flux and temperature of forest (A) and arable (B) soils at different soil moisture during freezing-thawing cycles.

Due to the measurement of CO_2 _flux in two hours intervals, we described the dynamics of CO_2 _emissions during repeated FTC in a detailed way. Although the initial levels of CO_2 _production were different in the forest and arable soils, the patterns of CO_2 _emission were generally similar for soils studied during both FTCs (Fig. 1). When the soil temperature decreased from +10 to negative temperature during freezing, soil respiration rate reduced immediately. In arable soils (pH_H2O _= 7.9), the evident rise of CO_2 _emission (from 1.0–2.8 to 5.6–6.9 mg C m^-2^h^-1^) have been observed soon after starting of freezing. This effect was very weak in the forest dry soil (FDS) and was not observed in the forest wet soil (FWS, pH_H2O _= 4.3–5.6).

The CO_2 _flux from the totally frozen soils never reached zero. During each thawing period we registered a sharp and two-levelled increase of CO_2 _emission. It started soon after soil thawing and lasted 1–2 days (Fig. 1). The CO_2 _emission rate increased up to 75 mg C m^-2^h^-1 ^in the forest soils and to 28 mg C m^-2^h^-1 ^in the arable soils during the first thawing. The acceleration of CO_2 _emissions lasted about 1–2 days; the subsequent decrease of the soil respiration was very slow. Before the second FTC, the CO_2 _emission level was higher than the CO_2 _emission levels at the beginning of the experiment. There was close correlation between CO_2 _emission rate and soil temperature during the freezing and thawing. Linear and polynomial models described these relationships satisfactory. The determination coefficients (R^2^) were 0.86–0.97 for linear regressions (P < 0.001) and changed between 0.94 and 0.99 (P < 0.001) for polynomial models.

### Carbon dioxide emission rates during different periods of FTC

We calculated mean values of CO_2 _fluxes and relative CO_2 _emission rates during different periods of freezing-thawing cycles (Table 1, 2). The emission rate at +10°C was taken as control level. Carbon dioxide emissions from soils at +10°C changed from 7.4 to 43.8 mg C m^-2^day^-1 ^and depended on the soil moisture and land use. The CO_2 _emission rates of forest soils with the higher contents of microbial biomass, soluble and total soil organic matter (SOM), were 2–3 times higher than SOM-poor agricultural soils. We observed the depressive effect of high soil moisture on respiration of arable soils: CO_2 _flux from arable wet soil (AWS) was twice less than from arable dry soil (ADS). In the forest soils, the depressive effect of high moisture on CO_2_emission was not observed. There is a close positive linear correlation between the initial CO_2 _emission rate and total C content in the topsoil (R^2 ^= 0.96; P < 0.01).

**Table 1:** The mean CO_2 _emission rates (mg C m^-2^h^-1 ^and STD in square brackets) of the soils during different periods of freezing-thawing cycles

Cycle	Period	Variants of soils			
		
		FDS	FWS	ADS	AWS
First cycle	Before freezing	35.1 [0.7]	43.8 [0.5]	14.6 [0.8]	7.4 [0.6]
	Soil freezing	12.3 [4.1]	14.1 [5.8]	2.7 [2.5]	4.6 [1.9]
	Frozen soils	4.5 [1.5]	3.7 [1.9]	0.8 [0.4]	1.1 [0.5]
	Soil thawing	24.6 [12.7]	27.8 [11.6]	10.1 [7.3]	2.6 [1.3]
	After thawing	54.7 [8.3]	58.3 [10.1]	25.1 [2.0]	11.4 [0.9]
					
Second cycle	Before freezing	43.3 [1.6]	55.1 [2.2]	19.6 [2.0]	10.2 [1.2]
	Soil freezing	11.1 [5.6]	15.9 [6.6]	3.9 [2.1]	6.8 [1.2]
	Frozen soils	2.7 [0.8]	2.5 [1.8]	0.8 [0.3]	1.5 [1.0]
	Soil thawing	15.5 [5.9]	15.2 [7.9]	8.4 [5.7]	1.4 [0.5]
	After thawing	41.0 [6.8]	42.6 [9.5]	19.0 [2.1]	8.8 [6.1]

**Table 2:** The relative CO_2 _emission rate of soils (part from initial rate at +10°C) during different periods of freezing-thawing cycles

Cycle	Period	Variants of soils			
		
		FDS	FWS	ADS	AWS
First cycle	Before freezing	1.00	1.00	1.00	1.00
	Soil freezing	0.35	0.33	0.18	0.63
	Frozen soils	*0.13*	*0.08*	*0.05*	*0.15*
	Soil thawing	0.70	0.63	0.70	0.35
	After thawing	**1.56**	**1.33**	**1.72**	**1.55**
					
Second cycle	Before freezing	1.23	1.26.	1.35	1.39
	Soil freezing	0.31	0.36	0.27	0.93
	Frozen soils	*0.08*	*0.06*	*0.06*	*0.20*
	Soil thawing	0.44	0.35	0.58	0.19
	After thawing	**1.17**	**0.97**	**1.30**	**1.19**

The respiration rate of completely frozen soil varied from 0.8 to 4.5 mg C m^-2^h^-1 ^and constituted 5–20% of the CO_2 _flux at +10°C. It indicates that microbial community was still active at -5°C and hence, evidences the existence of winter CO_2 _fluxes from tundra and boreal soils. During freezing and thawing, when the mean soil temperature was about 0°C, the CO_2 _emission rate varied strongly and depended on soil moisture and land use. Thus, CO_2 _fluxes from FDS, FWS and ADS amounted to 18–36% from initial respiration rate during freezing and were higher (35–70%) during thawing (Table 2). This pattern was reverse in AWS: respiration rate reached 63–93% of the initial level during freezing and constituted 19–35% during thawing. This contradiction may be explained by discovered increase of CO_2 _emission rate in arable soils immediately after starting of freezing. The mean values of CO_2 _emission after the first thawing were 1.3–1.7 times higher than before starting of freezing (Table 2). This difference was smaller after the second thawing and did not exceed 30%.

### Total and extra CO_2 _fluxes during FTCs

The total CO_2 _flux during two FTCs changed from 3.2 to 14.4 g C·m^-2^, the order of precedence was: AWS < ADS < FDS < FWS. These fluxes were approximately equal during the first and second FTCs (Table 3). There was a close positive correlation between the total CO_2 _flux values, total C content in the topsoil (0–4 cm) and WFPS of 0–4 cm and 4–8 cm layers (R^2 ^= 0.77 to 0.90; P < 0.01).

**Table 3:** Total and extra CO_2_-C flux (g C m^-2 ^period^-1^) during FTC

Cycle	CO_2_-C flux	Variants of soils			
		
		FDS	FWS	ADS	AWS
First cycle	**Extra flux (after thawing)**	**1.37**	**0.81**	**0.70**	**0.25**
	Total flux	7.00	7.34	2.73	1.47
	*Extra flux/Total flux, %*	*19.6*	*11.1*	*25.5*	*17.2*
					
Second cycle	Extra flux (before freezing)	0.23	0.32	0.14	0.08
	Extra flux (after thawing)	0.56	-0.10	0.41	0.13
	**Total extra flux**	**0.79**	**0.22**	**0.56**	**0.21**
	Total flux	6.46	7.04	2.84	1.77
	*Extra flux/Total flux, %*	*12.3*	*3.1*	*19.6*	*11.7*
					
The whole period	**Total extra flux**	**2.17**	**1.03**	**1.25**	**0.46**
	Total flux	13.5	14.4	5.56	3.24
	*Extra flux/Total flux, %*	*16.1*	*7.2*	*22.5*	*14.2*

The sum of extra CO_2 _fluxes induced freezing-thawing processes varied through 0.5–2.2 g C·m^-2^. The largest amount of extra CO_2 _emission was found in the FDS and the smallest one was observed in the AWS. The FWS and ADS provided the approximately equal extra CO_2 _fluxes: 1.0 and 1.3 g C·m^-2^. The freeze-thaw-induced CO_2 _emissions from forest soils were 2–3 times higher during the first FTC than for the second one. The extra CO_2 _fluxes from arable soils did not differ significantly during 1-st and 2-d FTC (Table 3). We found the significant negative correlation between the total extra CO_2 _fluxes and WFPS in 0–4 and 4–8 cm layers (R^2 ^= -0.81; P < 0.01). The share of extra CO_2 _fluxes in the total CO_2 _fluxes changed from 7.2 to 22.5% (Table 4). It was most significant in ADS (lowest level of initial CO_2 _emission), and was smallest in FDS (highest initial carbon dioxide emission).

**Table 4:** Chemical and microbiological properties of the forest and arable soils

Site	Soil layer	Statistics	pH (H_2_O)	Total, %		K_2_SO_4 _extraction, mg kg^-1^				MB*	
				
				C	N	C	N	NH_4_-N	NO_3_-N	C	N
FOREST	Litter, 2–3 cm	mean	5.6	29.3	1.0	898	90	32.8	4.7	nd	nd
		STD	0.3	5.5	0.2	436	40	21.7	3.5	nd	nd
	Humus layer, 0–4 cm	mean	4.3	3.4	0.2	32.6	3.1	0.5	0.2	84	9.2
		STD	0.1	1.2	0.1	17	1.4	0.2	0.2	43	7.9
	Mineral layer, 4–8 cm	mean	4.3	1.1	0.1	22	1.2	0.2	0.0	9.8	1.3
		STD	0.1	0.2	0.0	4	0.3	0.1	0.1	3.9	0.4
ARABLE	0–4 cm	mean	7.9	1.2	0.1	5	1.1	0.0	0.7	50	6.3
		STD	0.1	0.1	0.0	1	0.2	0.0	0.2	4.7	0.7
	4–8 cm	mean	7.9	1.2	0.1	6	1.7	0.0	1.2	71	8.0
		STD	0.1	0.1	0.0	1	0.3	0.0	0.3	14	0.7

STD – standard deviation, 5 replications; * – C and N immobilized in microbial biomass (MB), mg kg^-1^; nd-not determined

## Discussion

The results of our experiment well agree with the observations of Willis *et al. *[42]. They also found that the dry soils freeze faster and deeper than moist ones. The wet soils with the higher specific heat capacity required more energy consumption for cooling and subsequent freezing. The heat conductivity of mineral soils has been also found to be approximately an order higher than the heat conductivity of dry peat [43]. In our experiment, the freezing rate of arable soils with a low C content was also higher than in forest soil due to insulating function of the humus layer.

We observed two uncommon phenomena during our laboratory study: (1) the significant increase of CO_2 _emission from AWS soon after starting of freezing and (2) two-levelled pulse of CO_2 _emission during the thawing. We suppose that the first phenomenon may be caused by the higher dissolution of CO_2 _in the water phase of arable weak-alkaline soils during cooling and its subsequence release during freezing [44]. This physicochemical process takes place only in neutral and alkaline soils: HCO_3 _^- ^+ OH^- ^⇔ CO_2 _↑+ H_2_O. The evident acceleration of CO_2 _emission was also observed immediately after starting of freezing in the laboratory experiment with sterilized cultivated soils (pH = 6.8–7.2) [45]. It was shown that the sterilized weak-alkaline cultivated soil emitted 0.22–0.35 mg C-CO_2 _per 1 kg of soil during the freezing process. At the same time in sterilized forest soil with acidic reaction, the freezing-thawing events did not influence the dynamics of CO_2 _dissolution and emission. In field conditions, Zimov *et al. *[7,8] observed similar increase of CO_2 _emission rate during freezing of moist tundra soils due to physical release of trapped CO_2 _from soil pores and frozen water.

The two-levelled CO_2 _emission peak observed during soil thawing may be explained by two different processes. The first CO_2_-pulse lasted a short time and was governed by physical release of trapped CO_2_. The second one was higher and longer and was caused by an acceleration of microbial activity due to the temperature increase. This CO_2_-pulse possibly ensured from the organic substrates releases caused by microbial death during freezing [21,27,30,32,46]. Thus, it was found that thawing of soils produced an initial pulse (< 24 hours) in microbial respiration and that the total amount of carbon respired in each thaw period was largest during the first cycle and decreased in successive cycles [21,27]. We also observed similar decrease in surplus of CO_2 _emission and extra CO_2 _fluxes during the second FTC in comparison with the first one (Table 1 and 3). Schimel and Clien [21] believe that total respiration over the first cycle appears to dominate by the flush from microbial biomass, while the respiration over the second and next FTC is driven by the reduction in attack on the soil organic matter resulting from a reduced microbial population. It was found, that freezing-thawing induces stronger destabilization of microbial communities and it takes more time to restore in comparison with the drying of soils [46]. The restoration of frost-damaged microbial biomass takes about two weeks.

Our investigation showed that the respiration rate of completely frozen soils never reached zero indicating that the microbial community was still active at -5°C. These results well agree with other laboratory studies. The CO_2 _production of permanently frozen soils from northern regions has been found to remain positive and measurable at -16°C and even though -39°C [4,11]. The winter CO_2 _emission to atmosphere were also observed in a number of tundra and forest ecosystems under the field conditions [6-16,47]. The cold-season emission of CO_2 _from northern soils was a significant source of atmospheric CO_2 _that can account for up to half of the annual CO_2 _flux from Arctic and boreal forest ecosystems. The mechanism for the observed cold-season CO_2 _emission is not absolutely clear and could presumably result from variety of processes [1,7-9,19,25,48]: (1) the physical release of summer accumulated gases, (2) the biological activity occurring in a warm unthawed soil layer; (3) the microbial metabolism in unfrozen water films on the surface of soil particles; (4) the respiration of cold-adapted microbes and plant roots within the bulk of frozen soil.

We also showed that respiratory activation of forest soils induced by freezing-thawing was higher compared to arable soils. Such a different response of arable and forest soils to the repeated freezing-thawing events is governed by the differences in substrate availability, the size of microbial pool and root mass in soils. Thus, soil fungi dominating in forest soils have been considered the main source of CO_2 _release at low temperatures [1,4]. They are the cold-tolerant organisms and display a wider temperature range of metabolic activity than bacteria [49]. Besides, forest soils contained some amount of fresh leaves and roots (living and dead), while these materials were absent in arable soil. Therefore, the more significant acceleration of CO_2 _emission in forest soil during thawing could be caused by decomposition of carbon sources liberated from frost-damaged fresh organic materials [20,27].

## Conclusion

Our results showed the importance of soil moisture and land use on freeze-thaw-induced CO_2 _emission. These factors governed the initial rate of soil respiration, duration of freezing and thawing of soil, pattern of CO_2 _dynamics, and extra CO_2 _flux. As a rule, the freezing-thawing-induced emissions of CO_2 _were more significant in dry soils and during the first FTC. The forest soils demonstrated high respiratory activity at low temperatures and the more significant acceleration of CO_2 _emission during the thawing due to the predominance of fungi in microbial biomass and C-release from frost-damaged fresh organic matter. The cold soil respiration and acceleration of CO_2 _emission during the freezing-thawing was caused by different processes: the liberation of nutrients upon the soil freezing, biological activity occurring in unfrozen water films, and respiration of cold-adapted microflora. Since the cold season CO_2 _emission is mainly caused by the microbial activity (with some possible contribution of roots), our results can be used to develop the mathematical model of future winter CO_2 _emission under a warmer climate. The mechanisms of freeze-thaw activation also need future clarification.

## Methods

### Soils

The soil monoliths (diameter -15 cm, height 10–12 cm, weight 2.5–3.1 kg) were taken in October, 1999 from a beech forest and arable site (winter barley) both located in the central part of Germany (52° 30'N, 9°55'E). The field moisture of soils corresponds to 44% and 61% of WHC for forest and arable soils, respectively. The intact soil cores were adjusted to two contrast levels of water content: 65% of their water holding capacity (WHC) – dry soils and 100% of WHC – wet soils. To reach 100% of WHC moisture, the soil monoliths were placed in the tank with water. The water level in tank was equal to the height of monoliths. After 1–2 days, when soil cores were completely saturated with water, they were replaced in another tank for trickling the gravitational (free) water. To reach the soil moisture corresponding the 65% of WHC, we drained off the odd water from soil monoliths at 100% of WHC by means of a special vacuum-pumping devise. It was connected to the bottom of soil cores and kept the constant negative pressure corresponding to soil moisture 65% of WHC. This procedure allowed us to wet monoliths uniformly. The number of replications was five for each of four variants: (1) – Forest Dry Soil (FDS); (2) – Forest Wet Soil (FWS); (3) – Arable Dry Soil (ADS); (4) – Arable Wet Soil (AWS),

The forest and arable soils (Luvisols) were characterised by similar texture (loamy silt). The chemical properties of soils depended on the land use (Table 4). The pH of the forest and agricultural soil was 4.3 and 7.9, respectively. The humus layer of forest soils was characterised by higher content of total and dissolved carbon and nitrogen compared to arable topsoil. The C and N contents in microbial biomass were also higher in the forest soils.

### CO_2 _emission measurements

The automated gas chromatographic systems equipped with a ^63^Ni electron-capture detector [50] was used for CO_2 _emission measurements. The laboratory experiment was carried out in the microcosm systems [51] with intact soil monoliths, which allowed us to measure the CO_2 _fluxes from soils every two hours.

### Freezing-thawing cycles

Twenty soil columns were placed in the freezer and incubated at about +10°C during 4–5 days until the CO_2 _emission reached the constant level and then subjected to two FTC. To control the temperature in the freezer, the temperature sensors with automatic data logging were installed into the soil monoliths. There were 5 temperature sensors in each column. They were inserted on 1, 5, and 10 cm depths at the centre of monoliths, and 1, 5 cm depths near the side of monoliths. The temperature of soils was measured in one hour intervals with the data loggers. For our calculations, we used the mean values of soil temperature, since 5 sensors in each column demonstrated the similar temperature during experiment. To simulate freezing and thawing events in the soils the temperature in the freezer was changed from +10°C to -5°C during the first FTC and to -3°C during the second FTC. The initial level of CO_2 _fluxes at +10°C was a control to check the effect of FTC on CO_2 _emission. The duration of each freezing-thawing cycle was about 14 days. For future calculations we divided each FTC into five different periods depending on soil temperature (ST): (1) – before freezing, ST = +10°C; (2) – soil freezing, ST changed from +10 to -5°C; (3) – constant freezing temperature, ST = -5°C (for 1-st FTC) and ST = -3°C (for 2-d FTC); (4) – soil thawing, ST changed from -5 to +10°C; (5) – after thawing, ST = +10°C.

### Chemical and microbiological analysis of the soils

In the end of experiment each monolith of forest soil was divided into two layers: 0–4 cm (humus) and 4–8 cm (mineral). The forest litter was analysed separately. Arable cores were divided identically. The soil moisture, WHC and WFPS were determined by conventional methods of soil physics. The content of total C and N were determined using CN-analyser. Water soluble organic carbon, NO_3 _^- ^and NH_4 _^+ ^concentrations were measured colorimetrically in 0.5 M K_2_SO_4 _extracts (TRAACS 800 auto-analyser). Microbial C and N were determined by fumigation-extraction method and calculated as a difference between C and N contents in 0.5 M K_2_SO_4 _extracts before and after fumigation procedure. To estimate the amount of C immobilized in soil microbial biomass, we used k_EC _= 0.51 and k_EC _= 0.42 for forest and arable soils, respectively [52]. To calculate the amount of N immobilized in soil microbial biomass, we used k_EN _= 0.54 both for forest and arable soils [53]. We did not determine microbial C and N in forest litter since fumigation-extraction method was not applied for determination of microbial biomass in fresh organic materials [54].

### Data analyses and statistics

The mean CO_2 _emission rates (per hour) and fluxes (per period) were calculated for 5 different periods of each FTC. The extra CO_2 _fluxes (CO_2 _fluxes induced by freezing-thawing) were estimated for each period of FTC according to the following equations:

EFi = (Fi - Fo)* Di     (1)

where EFi is extra CO_2 _flux for i-period of FTC (mg C m^-2^period^-1^); Fi is mean CO_2 _flux during the i-period of FTC (mg C m^-2^day^-1^); Fo is mean CO_2 _flux at +10°C (before freezing, mg C m^-2^day^-1^); Di is duration of the i-period, days.

The results presented are arithmetic mean and standard deviation (STD). The correlation analysis was carried out using linear regression function. Statistical differences between treatments were tested by Student's T-test.

## List of abbreviations used

FTC – freezing-thawing cycle

WHC – water holding capacity

WFPS – water filled pore space

ST – soil temperature

SOM – soil organic matter

EF – extra CO_2 _flux

TEF – total extra CO_2 _flux

FWS – forest wet soil

FDS – forest dry soil

AWS – agricultural wet soil

ADS – agricultural dry soil

## Authors' contributions

IK conceived of the study, carried out the freezing-thawing experiments, made the chemical and microbiological analyses of soils, and wrote the manuscript. RT conceived of the study, and participated in its design, coordination and interpretation of results. LN realised the technical support of equipment, carried out the freezing-thawing experiments, and participated in results interpretation. All authors read and approved the final manuscript.
